# The role of digital inclusive finance in green innovation

**DOI:** 10.1371/journal.pone.0315598

**Published:** 2024-12-23

**Authors:** Cheng Chen, Min Fan, Yaojun Fan

**Affiliations:** 1 Chinese International College, Dhurakij Pundit University, Bangkok, Thailand; 2 School of Economics, Lanzhou University, Lanzhou, China; Al Ain University, UNITED ARAB EMIRATES

## Abstract

As environmental issues become more acute, green innovation has become a key driver in advancing environmental sustainability and a comprehensive green transition, paving the way towards a future of ’clear waters and blue skies’ and enhanced environmental quality. In this vein, financial support is deemed an important facilitator of green innovation. Nonetheless, traditional financial institutions often restrict investment in such projects due to biases surrounding the returns of green projects and difficulties in risk assessment. The rise of digital inclusive finance offers new insights into addressing this challenge. Drawing on data from Chinese A-share listed companies between 2014 and 2019 and employing a multidimensional fixed-effect model, this paper systematically investigates the impact and mechanisms of action of digital inclusive finance on corporate green innovation. The study finds that digital inclusive finance significantly spurs green innovation within enterprises; this finding remains robust following a series of robustness checks and the addressing of endogeneity concerns. The facilitative effect is more pronounced in non-state-owned enterprises and areas with stricter environmental regulations. Digital inclusive finance supports green innovation mainly through two pathways: ’funding effects,’ related to easing financing constraints and reducing transaction costs, and ’responsibility effects,’ pertaining to the enhancement of corporate social responsibility. Additionally, green innovation driven by digital inclusive finance further improves the quality of the ecological environment, leading to increased total factor productivity and overall corporate performance. This paper enriches the externalities research of digital inclusive finance, providing theoretical foundations and practical insights to foster the synergistic development of digital inclusive finance and green innovation.

## 1 Introduction

The continuous growth of finance undeniably stimulates economic development. Corporate leaders utilize credit avenues to enhance purchasing power, innovate production organization, and optimize resource distribution, thereby significantly improving resource allocation efficiency. This not only catalyzes the advanced evolution of industrial structures but also robustly propels comprehensive economic prosperity. Inclusive finance, introduced by the United Nations in 2005 and actively supported by the UN and the World Bank, has become a prominent trend in the financial sector. In 2015, China’s State Council unveiled the "Development Plan for Inclusive Finance (2016–2020)", clarifying its profound essence. Guided by principles of commercial sustainability and equal opportunities, it emphasizes policy guidance and support, strengthens the financial system framework, refines financial infrastructure, and provides effective financial services to all social strata, ensuring affordability. In this drive, China has notably emerged as a global leader in the rapid development of digital inclusive finance.

Digital finance, representing the innovative convergence of the internet and finance, is a cornerstone in China’s fintech strategy. Utilizing data analysis and information processing, it significantly enhances the quality and coverage of financial services, greatly increasing accessibility and unlocking economic growth potential, leading to higher income levels [1]. Numerous studies confirm the significant contribution of digital inclusive finance to economic growth [2]. It not only promotes inclusivity, helping to narrow the urban-rural income gap [3], but also benefits a wider population [4], continuously advancing poverty reduction efforts [5]. Additionally, the rise of digital inclusive finance strongly supports corporate growth. It fosters corporate innovation and expansion [6], drastically reduces financing costs for micro-enterprises [7], and accelerates capital structure optimization for SMEs [8]. Xie and colleagues [6] demonstrated through in-depth research that the development of digital inclusive finance lays a solid foundation for corporate innovation, significantly driving its growth. Moreover, the digital nature of digital inclusive finance expands the breadth and depth of financial services. Notably, the rapid rise of digital inclusive finance acts as a powerful catalyst for industrial digitalization, facilitating the integration of digital technologies with the real economy, and heralding transformative impacts on economic development and upgrades [9].

In response to global calls for climate action, China, in its role as a major nation, outlined its "dual carbon" blueprint at the 75th United Nations General Assembly, aiming for carbon dioxide emissions to peak by 2030. Since then, the Chinese economy has been transitioning from rapid growth to high-quality development, with "Building a Beautiful China" as a significant strategic objective. In this transition, integrating green development with economic growth becomes pivotal, potentially resolving the dichotomy between economic progress and environmental protection [10]. Green innovation serves as a powerful means for businesses to reap economic benefits and reduce ecological impact throughout a product’s lifecycle, achieving positive environmental outcomes [11]. It becomes a central force propelling high-quality societal development. Hence, exploring how digital inclusive finance influences green innovation holds profound implications, aiding in ecosystem restoration and advancing carbon peaking and neutrality goals.

As the concept of sustainable development deepens, it increasingly integrates with modern finance in multiple dimensions. In this context, finance, as a core element of green technology innovation, becomes crucial in directly affecting the effectiveness of corporate green technology innovation [12]. Currently, many scholars have conducted in-depth research on the impact of digital inclusive finance on green innovation. Several studies have found that the development of digital inclusive finance can significantly promote enterprises’ green technological innovation [13–15] and the output of green patents [16]. In this process, alleviating financial constraints on enterprises is a key way for digital inclusive finance to exert its effect [13, 17]. Additionally, digital inclusive finance can stimulate green technology innovation through mechanisms such as encouraging green consumption [17] and optimizing industrial structure [15]. Beyond the corporate level, digital inclusive finance plays an important role in regional green development. Research shows that digital inclusive finance helps to improve the green total factor productivity of the agricultural sector [18, 19], and also positively affects regional inclusive green growth [20] and urban green technological innovation [21]. Its mechanisms of influence include improving green technological innovation [20], optimizing energy structure [22], and more. Notably, the effect of digital inclusive finance on green innovation and the environment exhibits certain heterogeneity. Some studies have found that the promoting effect of digital inclusive finance on green innovation is more significant in eastern regions [21], high-income areas, and areas where agriculture is relatively concentrated [23]. Furthermore, the impact of different dimensions of digital inclusive finance (such as coverage breadth, usage depth, etc.) on green innovation also varies [14, 24].

Overall, while existing literature affirms the potential of digital finance to boost green development, gaps remain in systematically theorizing and rigorously verifying whether digital inclusive finance can fundamentally overcome the financing bottlenecks faced by green innovation and unleash the endogenous dynamics of environmental governance at the micro-level. This paper aims to systematically examine the relationship between digital inclusive finance and corporate green innovation, along with its micro-level impact mechanisms, based on existing studies. Specifically, the paper builds an econometric model using data from Chinese A-share listed companies from 2014 to 2019 to investigate the impact of digital inclusive finance on corporate green innovation and its heterogeneous characteristics. Further, it analyzes the mechanisms from the perspectives of alleviating financing constraints and enhancing corporate social responsibility to reveal the internal logic of how digital inclusive finance shapes the endogenous dynamics of corporate green innovation. Additionally, the paper assesses the impact of corporate green innovation on environmental performance and economic performance to comprehensively evaluate the externalities of digital inclusive finance. The conclusions not only deepen the understanding of micro-mechanisms in the field of financial technology but also provide practical implications for coordinating the development of digital inclusive finance and green innovation-driven strategies.

## 2 Theoretical analysis

Against the backdrop of China’s steady and rapid economic growth, the world has witnessed the remarkable "China Miracle." With the meteoric rise of the digital economy, digital inclusive finance has emerged as a significant opportunity for financial technologization. Today, after a series of evolutions and advancements, China’s digital inclusive finance stands at the forefront globally, setting a benchmark for the world. Its rapid development can be attributed to two primary factors: first, the supply shortage in the traditional financial sector, and second, lax regulatory policies, which have created a conducive environment for the growth of digital inclusive finance [25].

For the smooth advancement of green innovation activities, adequate funding for corporate R&D is indispensable. Solutions in this regard primarily involve two dimensions: alleviating financing constraints for businesses and reducing operational costs. With the aid of advanced digital technologies like big data, cloud computing, and mobile internet, China’s inclusive finance sector continues to make innovative strides, significantly reducing operational costs for financial institutions [7]. Importantly, digital inclusive finance, leveraging state-of-the-art digital technologies, can accurately identify customer needs and establish a robust third-party credit system. This effectively alleviates information asymmetry between SMEs and financial institutions, reducing financial mismatch risks [26] and business financing dilemmas [27].

In reality, financial market operations are often less than ideal, with pronounced information asymmetries between external investors and business managers. External investors cannot grasp the actual status of business investment projects as comprehensively as business managers, creating opportunities for managers to pursue personal gains, sometimes to the detriment of external investors [28]. This information asymmetry and potential game dynamics introduce financing constraints for business investments. Particularly for business innovation activities characterized by long-term horizons and uncertainties, financing constraints become even more pronounced, often relying solely on internal funding channels [29]. SMEs and other long-tail customer groups in traditional financial markets often face heightened financing constraints, mainly because conventional financial markets, due to cost and technological limitations, cannot efficiently serve these segments, resulting in market inefficiencies [30]. Excessive financing constraints not only elevate the default risk of SMEs [31] but also hamper their green innovation endeavors.

Fortunately, the rise of digital inclusive finance offers potential solutions. It breaks the shackles of traditional finance, emerging as a critical remedy for business financing challenges. On one hand, digital inclusive finance leverages advanced technologies to mine both standardized and non-standardized data, reducing information asymmetry between fund suppliers and seekers, and averting adverse selection and moral hazard. On the other hand, utilizing big data and other novel technologies, it reshapes the traditional financial system, innovatively changing traditional credit pricing models [32], thereby reducing market frictions, driving banking sector transformation, and enhancing the accessibility of financial services. Through internet platforms, digital inclusive finance can more conveniently improve businesses’ financing conditions, heightening information exchange and regulatory efficiency, and effectively circumventing future financing constraint predicaments [33]. When a significant barrier to green innovation—financing constraints—is alleviated, businesses will be more empowered to engage in green innovation activities. Digital inclusive finance stands as a potent tool to achieve this objective.

Undoubtedly, digital inclusive finance presents a sustainable opportunity by constructing a bridge in a responsible and economically viable manner, enabling all social strata, especially vulnerable groups and SMEs not fully covered by the current financial system, to access comprehensive, convenient, and continuous financial products and services equitably and effectively. The rise of mobile payment technology breaks the constraints of time and space, greatly enhancing the efficiency of goods and services transactions and reducing transaction costs for both parties. It not only addresses the high transaction fees resulting from banking system disparities but also mitigates risks associated with lost funds and counterfeit currency, subsequently reducing business transaction costs [34]. These cost reductions significantly increase companies’ surplus funds, effectively alleviating cash shortages and easing constraints on green innovation investments [35]. Thus, by alleviating financing restrictions and reducing costs, digital inclusive finance meets the capital requirements for green innovation, promoting its realization.

Moreover, the growth of digital inclusive finance undeniably serves as a powerful catalyst for the digital transformation of enterprises, providing a comprehensive and forward-looking roadmap for businesses’ digital metamorphosis. Mobile payment services in digital finance are reshaping business models. Leveraging apps like Alipay, WeChat, and Cloud QuickPay, a commercial ecosystem encompassing transport, education, and healthcare is being constructed, fostering emerging business models like shared mobility, digital education, and digital healthcare, offering new digital opportunities for enterprises [36]. Digitalization not only amplifies a company’s information processing capability, promoting data and knowledge flow and sharing, but also enables businesses to swiftly adapt to a fluctuating environment, enhancing supply chain flexibility. Digitally adept enterprises can effectively process and output structured and standardized information, optimizing information utilization, reducing information asymmetry with external financiers, and alleviating financing pressures [37]. Additionally, real-time monitoring of production status via smart programs facilitates the timely identification and resolution of equipment issues, reducing maintenance costs and time losses. Consequently, the digital transformation of enterprises is also a significant avenue for propelling green innovation.

We will further explore the impact of digital inclusive finance on green innovation from the perspective of corporate social responsibility awareness. With the expansion of digital inclusive finance and acceleration of enterprise digitalization, companies increasingly recognize the importance of fulfilling social responsibilities, thereby enhancing their social responsibility performance [38]. Stakeholder theory suggests that a firm comprises contracts with various stakeholders. While acquiring economic resources, a company needs to reciprocate by fulfilling its social responsibilities [39]. On one hand, digital technology is reshaping traditional organizational structures, redefining member identities, and constructing a shared, symbiotic, dynamic, loosely-coupled ecosystem. It rationalizes compensation and reward systems [40], significantly enhancing employee satisfaction [41], thereby elevating corporate social responsibility levels [42]. On the other hand, digital technology promotes upgrades in areas such as internal controls, financial management, information disclosure, and technological innovation, enhancing firms’ capability and efficiency in performing social responsibilities [43]. In contexts where formal external financing environments are underdeveloped, informal systems largely compensate for the formal system’s absence, profoundly influencing individual micro-decisions [44]. To foster green innovation, companies need to rely not just on internal resources and knowledge but also to assimilate external talent and expertise. Fulfilling social responsibilities helps companies form broader and deeper relational networks [45], directing external knowledge flow to them, thus advancing green technological innovation endeavors. Corporate social responsibility, as a manifestation of a company’s contribution to society, has become a novel resource and capability, compelling companies to evolve in a more diversified, efficient, and green direction. Hence, from a corporate social responsibility perspective, digital inclusive finance plays a positive role in promoting green innovation.

Lastly, the diverse policies and measures that governments enact for ecological and environmental protection significantly impact enterprises’ production costs, resource allocation, and management efficiency. Environmental regulations, as a robust government strategy driving businesses towards green development, effectively shape the positive influence of digital inclusive finance on green innovation. Notably, under varying intensities of environmental regulation, enterprises exhibit distinct tendencies in green technological innovation, resulting in diversified effects of digital inclusive finance on green innovation [1]. As early as 1991, Porter introduced the influential "Porter Hypothesis", suggesting that appropriate environmental regulations can spur businesses to undertake green technological innovations, reduce costs, elevate product quality, and potentially bolster their competitiveness in international markets. In this context, the inclusive nature of digital finance aids in integrating numerous groups facing financing restrictions into the financial market, alleviating disparities between environmental governance costs and green innovation funding allocations. Additionally, it can expedite the pace of green innovations by expanding financing pathways, diminishing rent-seeking opportunities, easing financing constraints, and providing abundant funds to businesses [46]. Under high environmental regulation intensity, companies experience the "innovation compensation" effect, being incentivized to gain market share and profit through green technological innovations and construct market barriers [1]. Therefore, it can be asserted that under high environmental regulation intensity, the promotion of green innovation by digital inclusive finance is more pronounced.

In summary, we propose the following research hypothesis:

The burgeoning development of digital inclusive finance acts as a potent catalyst propelling green innovation.

## 3 Methodology

### 3.1. Sample selection and data sources

This study selects Chinese A-share listed companies and urban digital inclusive finance from 2014 to 2019 as research samples. We refined the sample selection according to the following procedure: ① Exclude companies in the financial sector; ② Exclude firms labeled as ST or *ST; ③ Exclude samples with missing primary variable data. After filtering, we obtained 14,220 company-year observations. To mitigate the impact of outliers, we performed a 1% winsorization on primary corporate variables with extreme distributions. The company-level data is sourced from the CSMAR database, while the city-level data comes from the "China City Statistical Yearbook" and CNRDS database.

### 3.2. Core variable definitions

#### 3.2.1. Dependent variable: Green Innovation (Inno)

Referring to the research method of Ma et al. (2021) [47], we quantify green innovation by the number of green patent applications submitted by the company in a given year. Compared to other potential indicators, the number of green patent applications or authorizations more directly and accurately reveals a company’s innovative strength [48]. Additionally, since patent technology can impact corporate performance at the application stage, patent application data is often more stable, reliable, and timely than authorization data [49]. Therefore, we chose the relatively stable, trustworthy, and timely count of patent applications as our metric. To remain consistent with previous research, we applied a logarithmic transformation to the number of green patent applications in a given year, which includes adding one to the count.

#### 3.2.2. Explanatory variable: Digital Inclusive Finance (DFI)

Our research relies on the digital inclusive finance index jointly constructed by Peking University’s Digital Finance Research Center and the Ant Group. This index has gained wide academic recognition and has been extensively applied in related studies [6, 50]. To facilitate data analysis, we divided the digital inclusive finance index by 100 and standardized it.

#### 3.2.3. Control variables

Based on the research of Ma et al. (2021) [47], we selected the following variables as control variables: executive shareholding ratio (Stock), board size (Board), company growth potential (Growth), company size (Size), company maturity (Age), executive compensation (Wage), dual roles (State), market influence (Power), return on total assets (Roa), liquidity ratio (LR), fixed asset turnover (Fix), capital concentration (Capi), equity concentration (Con), Tobin-Q value (TBQ), and economic development level (Eco).

As shown in Table 1, we have provided detailed definitions and explanations for each variable.

**Table 1:** Variable definition table.

Types of variables	Name of variables	Symbol of variables	Definition of variables
Explained variable	Green innovation	EPR	ln(Number of enterprise green patent applications +1)
Core explanatory variable	Digital Financial Inclusion	DFI	Peking University Digital Financial Inclusion Index
Control variables	Executive ownership ratio	Stock	Number of shares held by executives/total number of shares
	Board size	Board	The logarithm of the total number of directors of a corporation
	Growth ability	Growth	Current main business income/previous main business income
	Enterprise scale	Size	ln(Assets of Enterprise)
	Maturity of enterprise	Age	ln(Years on the market)
	Executive compensation	Wage	Take logarithm of the total salary compensation of the top three executives
	Duality	State	If the chairman and CEO are the same person, it is 1; otherwise, it is 0 and it is a dummy variable
	Market power	Power	ln(The ratio of operating revenue to operating cost)
	Rate of return on assets	Roa	Profit for the year/total assets at end of period
	Liquidity ratio	LR	Current liabilities/current assets
	Turnover of fixed assets	Fix	Operating income/Average fixed assets
	Capital-intensity	Capi	ln(The ratio of total fixed assets to the number of employees)
	Ownership concentration	Con	Number of shares held by the largest shareholder/total number of shares
	Tobin Q value	TBQ	Tobin Q value
	Economic development level	Eco	City night light data

### 3.3. Model design

Drawing on previous studies, this study constructs the following model to examine the relationship between digital financial inclusion and green innovation:

$$EPR_{i,t}=\alpha_{0}+\alpha_{1}DFI_{i,t}+\delta X+\gamma_{c}+\omega_{t}+\varepsilon_{i,t}$$ (1)

In Formula (1), subscript i is enterprise, c is industry, and t is year. The explained variable EPR is green innovation, the core explanatory variable DFI is the urban digital financial inclusion index, and X is the control variable. *γ*_*c*_ is an industry fixed effect, used to control the inherent differences between different industries. *ω*_*t*_ is a time-fixed effect, which is used to control the influence of unobservable factors over time on enterprises’ green innovation. As mentioned above, if digital financial inclusion promotes corporate green innovation, *α*_1_ in the expected model is significantly positive. The descriptive statistics of main variables are shown in Table 2.

**Table 2:** Descriptive statistics.

Variable	Obs	Mean	Std. Dev.	Min	Max
Inno	14223	0.5353	0.9397	0.0000	4.1431
DIF	14223	-0.0000	1.0000	-2.9443	1.8707
Stock	14223	13.1042	18.4543	0.0000	65.5639
Board	14223	2.3122	0.2174	1.7918	2.8904
Growth	14223	1.7130	2.8970	-0.1854	24.6177
Size	14223	22.3543	1.2543	20.0986	26.3245
Age	14223	2.4104	0.5849	1.3863	3.3322
Wage	14223	14.5137	0.6643	12.9480	16.4322
State	14223	0.2775	0.4478	0.0000	1.0000
Power	14223	0.9136	0.2009	0.6923	1.8682
Roa	14223	0.0311	0.0694	-0.3332	0.1878
LR	14223	2.8536	4.1529	0.2192	30.0732
Fix	14223	8.8028	21.0576	0.3078	163.5984
Capi	14223	12.5824	1.0826	9.4684	15.4415
Con	14223	33.3026	14.1374	9.0800	70.8600
TBQ	14223	2.1331	1.4050	0.8558	8.9352
Eco	14223	5.5852	5.7297	0.0042	22.1829

## 4 Results

### 4.1. Empirical analysis

The benchmark regression results are presented in Table 3. Columns (1) and (2) show the regression outcomes without and with control variables, respectively. The results indicate that digital inclusive finance positively influences corporate green innovation, confirming the hypothesis proposed in this study and aligning with existing literature [51, 52]. Digital inclusive finance aims to provide appropriate, effective, and affordable financial services to all societal levels and groups, based on the principles of equal opportunity and business sustainability, thereby meeting their financial service needs. To verify the inclusivity of digital inclusive finance, regression analyses were conducted specifically on enterprises within the SME and ChiNext sectors of China’s A-share market. If the hypothesis is validated, it would imply that digital inclusive finance also positively impacts the green innovation of smaller, less advantaged enterprises, rather than causing discriminatory effects. The regression results for the SME and ChiNext sectors, as shown in columns (3) and (4) of Table 3, are significant. This finding highlights the inclusiveness in the development of digital inclusive finance, consistent with the findings of Allen et al. (2016) [4].

**Table 3:** Empirical analysis.

	(1)	(2)	(3)	(4)
	Inno	Inno	Inno	Inno
DIF	0.0770***	0.0401**	0.0733*	0.1095**
	(0.0168)	(0.0197)	(0.0426)	(0.0536)
Stock		0.0001	0.0016*	-0.0018*
		(0.0005)	(0.0008)	(0.0010)
Board		0.0539	0.0993	-0.0136
		(0.0334)	(0.0711)	(0.0802)
Growth		-0.0090***	-0.0073	-0.0042
		(0.0024)	(0.0084)	(0.0082)
Size		0.2442***	0.2421***	0.2450***
		(0.0085)	(0.0214)	(0.0291)
Age		-0.0804***	0.0642	-0.1189*
		(0.0162)	(0.0509)	(0.0628)
Wage		0.0974***	0.1359***	0.1520***
		(0.0132)	(0.0272)	(0.0364)
State		-0.0216	-0.0228	0.0370
		(0.0161)	(0.0306)	(0.0355)
Power		-0.0938**	0.0763	-0.3862***
		(0.0466)	(0.1068)	(0.1002)
Roa		0.3598***	0.5402**	0.4691**
		(0.1110)	(0.2304)	(0.2293)
LR		-0.0106***	-0.0139***	-0.0003
		(0.0017)	(0.0036)	(0.0046)
Fix		-0.0017***	-0.0034***	-0.0016
		(0.0004)	(0.0010)	(0.0012)
Capi		-0.0504***	-0.0960***	-0.0168
		(0.0091)	(0.0198)	(0.0237)
Con		-0.0000	-0.0008	0.0004
		(0.0005)	(0.0011)	(0.0016)
TBQ		0.0159**	0.0126	-0.0026
		(0.0063)	(0.0142)	(0.0157)
Eco		-0.0019	0.0105***	-0.0012
		(0.0016)	(0.0033)	(0.0036)
_cons	0.5354***	-5.5165***	-5.9966***	-5.9556***
	(0.0072)	(0.2311)	(0.5495)	(0.7235)
Control	NO	YES	YES	YES
Industry_FE	YES	YES	YES	YES
Year_FE	YES	YES	YES	YES
Obs	14220	14220	3537	2364
r2_a	0.1595	0.2544	0.2310	0.2466

Note:

***, ** and * represent the significance level of 1%, 5% and 10% respectively.

### 4.2. Robustness test

To ensure the reliability and validity of our research findings, we conducted a series of robustness tests as detailed in our paper. Specifically, these tests included adopting a dependent variable replacement strategy, using the difference GMM method, introducing high-dimensional fixed effects, lagging control variables by one period, and employing the instrumental variable method. The results of these robustness tests consistently indicate that digital inclusive finance positively influences corporate green innovation. Detailed results and analyses of these tests are provided in the appendix for reference (See S1 File).

### 4.3. Mechanism analysis

Within the framework of our study, we drew inspiration from Wen and Ye (2014) [53] for their revision and innovation of Baron and Kenny’s (1986) stepwise analytical method, constructing the following mediation effect test model. Here, Eq (2) aligns with Eq (1), functioning as the mediating variable.


$$EPR_{i,t}=\alpha_{0}+\alpha_{1}DFI_{i,t}+\delta X+\gamma_{c}+\omega_{t}+\varepsilon_{i,t}$$ (2)

$$Chan_{i,t}=\alpha_{0}+\alpha_{1}DFI_{i,t}+\delta X+\gamma_{c}+\omega_{t}+\varepsilon_{i,t}$$ (3)

$$EPR_{i,t}=\alpha_{0}+\alpha_{1}DFI_{i,t}+\alpha_{2}Chan_{i,t}+\delta X+\gamma_{c}+\omega_{t}+\varepsilon_{i,t}$$ (4)

#### 4.3.1. Funding effect

Our theoretical analysis initially revealed that the rise of digital inclusive finance helps enterprises effectively alleviate financing difficulties, paving the way for green innovation. To explore the potential impact of digital inclusive finance on corporate financing constraints, we utilized the SA index, constructed based on two relatively stable and highly exogenous variables—firm size and years since listing—to measure the extent of financing constraints faced by enterprises. The SA index is negative, and its absolute value represents the severity of financing constraints—the larger the value, the more severe the constraints. Columns (1) and (2) in Table 4 present the regression results of the mediation effect, confirming its existence with a calculated effect value of 0.0048. This finding further corroborates that the development of digital inclusive finance can significantly alleviate corporate financing constraints, ultimately fostering green innovation in enterprises. This conclusion is also supported by the literature, including the work of Zhong et al. (2022) [1].

**Table 4:** Mechanism analysis 1.

	(1)	(2)	(3)	(4)
	SA	Inno	Cost	Inno
DIF	-0.0224***	0.0391*	-0.0053***	0.0418*
	(0.0058)	(0.0228)	(0.0017)	(0.0228)
SA		-0.2185***		
		(0.0368)		
Cost				-0.4035***
				(0.1267)
Control	YES	YES	YES	YES
Industry_FE	YES	YES	YES	YES
Year_FE	YES	YES	YES	YES
Obs	11691	11691	11693	11693
r2_a	0.3094	0.2634	0.1405	0.2618

Additionally, our theoretical discussion suggests that digital inclusive finance can effectively reduce corporate expenses in transactions and other areas, thereby catalyzing the unfolding of green innovation in enterprises. Following Li’s (2022) [34] research, we chose the financial expense ratio (Cost) as an indicator of corporate costs. Using the aforementioned mediation effect testing procedure, we obtained the results shown in columns (3) and (4) of Table 4. These results confirm the existence of a mediation effect, with an effect value of 0.0021. This aligns with our previous analysis, indicating that digital inclusive finance breaks the constraints of time and space, enhances the efficiency of transactions and settlements of goods and services, reduces the operational burden of enterprises, and thus fosters green innovation in enterprises.

As an integral part of financial infrastructure, digital finance has provided unprecedented opportunities and conditions for corporate technological innovation [36]. Theoretical analysis indicates that digital methods can help companies solve financial and other issues, thereby promoting the implementation of corporate green innovation. Hence, we also include the degree of corporate digitalization as a mediating variable in our analysis. We measure the degree of corporate digitalization using the logarithm of a company’s intangible assets in digital technology (Num). The regression results of the mediating effect, as shown in columns (1) and (2) of Table 5, confirm the existence of a mediating effect, with an effect value of 0.0073. This finding is consistent with existing research literature—the rise of digital technology has stimulated consumers’ pursuit of product diversification, constructing a bidirectional interactive supply-demand relationship model [54], enabling more efficient matching of consumer demands with corporate green process research and development [55], thereby fostering the robust development of corporate green innovation.

**Table 5:** Mechanism analysis 2.

	(1)	(2)	(3)	(4)
	Num	Inno	Resb	Inno
DIF	0.2346***	0.0434**	0.9576***	0.0389**
	(0.0349)	(0.0221)	(0.2367)	(0.0197)
Num		0.0311***		
		(0.0060)		
Resb				0.0013*
				(0.0007)
Control	YES	YES	YES	YES
Industry_FE	YES	YES	YES	YES
Year_FE	YES	YES	YES	YES
Obs	11123	11123	14220	14220
r2_a	0.5224	0.2444	0.4180	0.2545

#### 4.3.2. Responsibility effect

To reveal how digital inclusive finance promotes corporate green innovation by strengthening corporate social responsibility, this study conducts an in-depth analysis using corporate social responsibility as a mediating variable. Although there are no fixed standards for measuring corporate social responsibility, diverse assessment methods—such as stakeholder comprehensive performance, third-party corporate social responsibility rating indexes, and social responsibility explicit issue methods [56]—provide a wealth of reference perspectives. In this research, we chose the overall corporate social responsibility score provided by Hexun.com for listed companies (Resb) as the standard for measuring corporate social responsibility performance. The mediation effect regression results in columns (3) and (4) of Table 5 indicate the mediating effect of corporate social responsibility, with a mediating effect value of 0.0012.

### 4.4. Applicability analysis

#### 4.4.1. Nature of enterprises

Initially, this research focuses on how enterprises of different ownership structures respond to digital inclusive finance. State-owned enterprises often enjoy more relaxed bank loan policies and have a clear advantage in accessing credit resources, so the role of digital inclusive finance in alleviating their financial issues might be less pronounced. In contrast, non-state-owned enterprises might benefit more from the development of digital inclusive finance, propelling them more vigorously towards green innovation.

To test this hypothesis, detailed regression analysis for enterprises of different ownership structures (marked as SOE) was conducted. The regression results displayed in columns (1) and (2) of Table 6 show that the effect of digital inclusive finance in promoting green innovation is more significant for non-state-owned enterprises. This not only validates our initial suppositions but also aligns with existing research, such as that by Qiao et al. (2022) [52].

**Table 6:** Applicability and economic consequence analysis.

	(1)	(2)	(3)	(4)	(5)	(6)	(7)
	Inno	Inno	Inno	Inno	ΔGreen	ΔTFP	ΔPerf
DIF	0.0618*	0.0730***	0.1009***	0.0187			
	(0.0363)	(0.0235)	(0.0298)	(0.0322)			
$\hat{\Delta {Inno}_{i,t}}$					0.0252**	1.3134***	1.3530***
					(0.0126)	(0.1889)	(0.3555)
Control	YES	YES	YES	YES	YES	YES	YES
Industry_FE	YES	YES	YES	YES	YES	YES	YES
Year_FE	YES	YES	YES	YES	YES	YES	YES
Obs	4991	9226	6283	6092	7630	7660	5819
r2_a	0.3455	0.2402	0.2631	0.2691	0.8710	0.0571	0.0571

#### 4.4.2. Environmental regulation

This section seeks to understand how the intensity of environmental regulations affects the green innovation ambitions of companies. It is anticipated that under more stringent environmental regulations, the promotive effect of digital inclusive finance on corporate green innovation will be more pronounced. Drawing from the work of Liu et al. (2021) [57], we adopted the proportion of completed investment in industrial pollution control in the secondary sector as an indicator to measure the strength of environmental regulation (denoted as ENV).

To test this conjecture, we constructed a dummy variable for the intensity of environmental regulation (DENV), determined by the annual ENV median of the industry in which the company operates. Based on this, we grouped the sample and performed regression analysis separately. Columns (3) and (4) of Table 6 reveal the regression outcomes, indicating that digital inclusive finance has a more pronounced effect in promoting corporate green innovation under higher environmental regulatory intensity. This finding is supported by existing literature, such as the research by Zhong et al. (2022) [1].

### 4.5. Ecological and economic consequence analysis

Numerous studies have shown that digital inclusive finance infuses the socio-economic landscape with diversity and vitality, significantly affecting the trajectory of the macroeconomy, income distribution patterns, corporate finance strategies, and household financial management [58, 59]. This paper delves deeply into the positive role of digital inclusive finance in catalyzing green innovation. We pose a pivotal question: How will the impetus of digital inclusive finance in stimulating green innovation activities shape the future ecological and economic landscape? To address this, we further analyze the consequences from the dual perspectives of ecological preservation and corporate prosperity.

#### 4.5.1. Ecological consequence analysis

The continual growth and evolution of green innovation signify our transition towards a greener and more sustainable economic system. Such a shift will mitigate the profound impacts of economic growth on the environment, giving the ecosystem more room for recovery and regeneration. Combined with our earlier analysis, we recognize that digital inclusive finance actively fosters green innovation through its financial pathways and responsibility effects, paving the way for sustained environmental improvement. To rigorously validate this logic chain, we drew on the research methodology of Kim et al. (2021) [60], employing a two-stage least squares model to accurately identify and quantify the specific impact of digital inclusive finance’s development in propelling corporate green innovation, as well as its substantive effect on the future ecological environment.

Specifically, in the first stage, the benchmark model is changed into the following difference model, and the fitting value of the regression variable Δ*Inno*_*i*,*t*_ represents the impact of the development of digital financial inclusion on the shift of enterprises’ green innovation.


$$\Delta Inno_{i,t}=\alpha_{0}+\alpha_{1}\Delta DFI_{i,t}+\delta \Delta X+\gamma_{c}+\omega_{t}+\varepsilon_{i,t}$$ (5)

In the second stage, we use the "Green Index" issued by the "China Green Development Index Report" to measure the ecological environment quality of each province in China. As shown in Eq (6), t+1 year green index Δ*Green* of each province is taken as the model dependent variable, and the fitting value of Δ*Inno*_*i*,*t*_ in the first stage is taken as the core explanatory variable for regression.


$$\Delta Green_{i,t}=\alpha_{0}+\alpha_{1}\hat{\Delta Inno_{i,t}}+\delta \Delta X+\gamma_{c}+\omega_{t}+\varepsilon_{i,t}$$ (6)

Table 6 (5) column shows the second stage regression results, the coefficient of $\hat{\Delta {Inno}_{i,t}}$ is 0.0252, significant at the 5% level. It indicates that with the development of digital inclusive finance, the green innovation level of enterprises has been improved, and thus the ecological environment has been significantly improved in the future. This confirms the logic and is supported by existing research.

#### 4.5.2. Economic consequence analysis

Green innovation is a proactive approach to incorporating an environmental perspective into strategic planning. It emphasizes pollution prevention and adopts sustainable development as a strategic goal. Through green innovation, companies can not only drive technological advancements but also mitigate the adverse environmental impacts of their production and business activities. Simultaneously, they can enhance total factor productivity (TFP), enabling enterprises to achieve more substantial economic benefits [61]. Resource-based theory further underscores that a firm’s competitive advantage stems from its unique combination of resources and capabilities [62]. A green image constitutes not only a unique resource for the enterprise but also a leveraged resource that can enhance other resources, significantly boosting corporate performance [63].

Hence, we anticipate that the continuous development of digital inclusive finance will stimulate the prosperity of green innovation, further propelling a comprehensive enhancement of enterprise TFP and performance. To test this hypothesis, we intend to employ a two-stage model to deeply explore how the growth of digital inclusive finance leads the transformation of corporate green innovation, subsequently exerting a substantive impact on future enterprise TFP and performance.


$$\Delta TFP_{i,t}=\alpha_{0}+\alpha_{1}\hat{\Delta Inno_{i,t}}+\delta \Delta X+\gamma_{c}+\omega_{t}+\varepsilon_{i,t}$$ (7)

$$\Delta Perf_{i,t}=\alpha_{0}+\alpha_{1}\hat{\Delta Inno_{i,t}}+\delta \Delta X+\gamma_{c}+\omega_{t}+\varepsilon_{i,t}$$ (8)

In Eqs (7) and (8), we employ the GMM method to estimate the Total Factor Productivity (TFP) of enterprises and measure corporate performance through the net operating profit margin. Column (6) of Table 6 reveals the second-stage regression analysis results related to enterprise TFP. Here, the coefficient is 1.3134, significant at the 1% level, clearly indicating that the expansion of digital inclusive finance significantly propels the growth of corporate green innovation, infusing potential and vitality for enterprises’ future expansion. Meanwhile, column (7) displays the second-stage regression analysis results concerning corporate performance, where the coefficient is 1.3530, significant at the 1% level, denoting that the evolution of digital inclusive finance significantly promotes the enhancement of corporate green innovation, laying a foundation for future economic benefits. This finding not only validates our earlier conjectures but also aligns with existing research conclusions.

## 5 Discussion

This study explores the role of digital inclusive finance in fostering green innovation. Empirical results demonstrate that digital inclusive finance significantly promotes the development of green innovation. Our findings highlight the importance of digital inclusive finance in promoting corporate green innovation, consistent with the results of Qiao et al. (2022) [52]. By offering broader and more accessible financial services, digital inclusive finance provides essential funding support for green innovation, especially for small and medium-sized enterprises (SMEs) that are often underserved by traditional financial services.

Our analysis of mediation mechanisms reveals that digital inclusive finance promotes green innovation through two main pathways: the funding effect and the responsibility effect. The funding effect is evident as digital inclusive finance directly provides necessary financial support and indirectly resolves funding issues by lowering transaction costs and facilitating digital transformation, particularly for SMEs. This aligns with literature suggesting that digital inclusive finance not only reduces financing costs but also spurs investment in green technology and projects by increasing fund availability [1, 64]. Conversely, the responsibility effect shows that digital inclusive finance strengthens corporate social responsibility, enhancing firms’ commitment to environmental stewardship alongside financial support. This finding reveals a significant phenomenon: digital inclusive finance robustly fosters the growth of corporate social responsibility, enabling firms to acquire more tangible and intangible resources. This not only broadens the scope of corporate social responsibility but also paves the way for green innovation, ensuring a sustainable path forward [65].

The study also reveals significant differences in the impact of digital inclusive finance on green innovation across different types of enterprises. While digital inclusive finance substantially drives green innovation, its impact varies between state-owned and non-state-owned enterprises, with a more pronounced effect on the latter. This is likely because non-state-owned enterprises face greater constraints in accessing traditional financial resources, making digital inclusive finance more crucial for them, as corroborated by existing research [52]. Additionally, market-based environmental regulations like emission trading schemes show that non-state-owned enterprises are more adept at seizing market opportunities for green innovation [48, 52]. Our research also finds that the impact of digital inclusive finance on green innovation is more significant in firms more affected by environmental regulations, supporting existing literature [1]. This underscores the pivotal role of environmental regulation in guiding firms toward green development. For companies with less stringent environmental regulations, digital inclusive finance might need to be coupled with stronger policy support to effectively promote green innovation.

In analyzing the ecological and economic consequences, we reveal that digital inclusive finance in promoting green innovation has significant positive impacts on both the ecological environment and corporate economy. On one hand, green innovation spurred by digital inclusive finance significantly improves the green index, enhancing future ecological conditions. This transformation mitigates the harsh impact of economic growth on the environment, providing more room for ecological restoration and regeneration. On the other hand, green innovation, as a proactive strategy integrating environmental considerations, profoundly impacts firms. It not only fosters technological advancement but also improves firms’ total factor productivity by reducing the negative environmental impact of their operations. This means that through green innovation, firms achieve environmental protection goals and reap greater economic benefits. Additionally, a green image, as a unique resource, adds value to the firm and, as a leveraged resource, significantly boosts overall performance. These findings align with the resource-based theory, emphasizing that a firm’s competitive advantage stems from the synergy of its unique resources and capabilities.

Compared to existing literature, this paper’s innovations are mainly reflected in the following aspects. Firstly, it comprehensively investigates the micro-mechanisms of how digital inclusive finance influences corporate green innovation. While most existing studies adopt a macro perspective, focusing on the role of digital inclusive finance in regional green development, this paper centers on the micro-level of enterprises. Through theoretical analysis and empirical testing, it reveals how digital inclusive finance impacts corporate green innovation decisions by alleviating financial constraints, reducing transaction costs, and enhancing corporate social responsibility, enriching our understanding of the externalities of digital inclusive finance. Secondly, it systematically examines the heterogeneous characteristics and influencing factors of digital inclusive finance’s impact on green innovation. By comparative analysis among different ownership structures, industries, and regions, the paper finds significant differences in the effect of digital inclusive finance on green innovation. It further reveals the role of factors such as enterprise financing needs, industry pollution levels, and regional resource endowments in forming heterogeneity, offering references for tailored, category-specific policy implementation that leverages digital inclusive finance’s supportive role. Thirdly, the paper explores the moderating role of environmental regulation in the process of digital inclusive finance impacting green innovation. In addition to testing the direct effects of digital inclusive finance on green innovation, the paper incorporates an environmental regulatory strength perspective for empirical analysis. It reveals differences in their relationship under diverse environmental regulatory contexts, highlighting the positive significance of government environmental governance in optimizing the resource allocation of digital inclusive finance. This enriches studies on the interaction between institutional factors and financial innovation. Fourthly, it assesses the ecological benefits and economic effects brought by digital inclusive finance through promoting green innovation. Existing literature may either explore the economic growth effect of digital inclusive finance or focus on its impact on the ecological environment, rarely considering both together. This study, upon clarifying the relationship between digital inclusive finance and green innovation, further empirically tests how their interaction affects enterprises’ resource utilization efficiency, pollutant emission intensity, and overall performance. It evaluates the comprehensive effect of digital inclusive finance within a unified analytical framework, which holds significant value for understanding the relationship between the digital economy and green development.

In summary, through a micro perspective and multidimensional examination, this paper not only enriches the study of the externalities of digital inclusive finance but also provides theoretical support and practical insights for promoting the green transformation of enterprises enabled by digital technology. The conclusions contribute to optimizing financial resource allocation by financial market participants, pushing for innovation in green financial products and services, and offering references for governments to use a combination of financial, industrial, and environmental policies to foster an institutional environment favorable for green innovation. This has critical practical significance for accelerating the construction of a green, low-carbon, and circular development economic system and advancing ecological civilization construction. Future research could further explore the dynamic relationship between digital inclusive finance and green innovation, as well as how to enhance this relationship through policy interventions and technological innovation. Given the urgency of global environmental change and the green economic transition, studying the application of digital inclusive finance in different countries and regions and its impact on green innovation will have significant international implications.

## 6 Conclusions and policy recommendations

### 6.1. Conclusions

This study explores the role of digital inclusive finance in enhancing green innovation. Through empirical analysis of A-share listed companies in China, we find that digital inclusive finance significantly positively influences corporate green innovation capability. This impact is also evident in SMEs and start-up board companies, demonstrating the inclusiveness of digital inclusive finance. These results not only confirm the importance of digital inclusive finance in promoting environmental sustainability but also highlight its role in compensating for the limitations of traditional finance in supporting green projects. Mechanism analysis reveals that digital inclusive finance can influence corporate green innovation through funding and responsibility effects. Applicability analysis finds that the impact of digital inclusive finance on green innovation is more pronounced in non-state-owned enterprises and those more affected by environmental regulations. In the analysis of ecological and economic consequences, we discover that the development of digital inclusive finance has significantly improved the future ecological environment and enhanced the total factor productivity and performance of enterprises. Our study offers a new perspective on understanding the role of digital inclusive finance in promoting green innovation. This not only enriches existing financial and innovation theories but also provides valuable insights for policymakers and practitioners.

### 6.2. Policy recommendations

Digital inclusive finance has demonstrated its remarkable power, facilitating robust economic development and significantly advancing poverty reduction. Based on this study and previous academic findings, we firmly believe that countries around the world should be more proactive in promoting the growth of digital inclusive finance to simultaneously enhance environmental and economic benefits. We offer the following policy recommendations. Firstly, vigorous efforts should be made to drive innovation in financial technology, strive to narrow the "digital divide," and utilize highly developed means of digital inclusive finance to promote high-quality economic development. For instance, by innovating service models, actively adopting cutting-edge technologies, and optimizing operational processes, more social capital can be mobilized to participate, facilitating more precise services for small and micro enterprises and socially vulnerable groups. Given that impoverished regions often lack sufficient human capital and knowledge of digital inclusive finance, this limits its penetration and efficacy. Therefore, governments should enhance the financial literacy of impoverished populations by incorporating internet and basic financial knowledge into compulsory education systems and strengthening the construction of rural educational infrastructure and support for education and training. Secondly, a system of fiscal support for digital inclusive finance’s role in poverty alleviation should be established and refined. In the event of uncertainty shocks, short-term financial incentives should be provided to digital financial institutions to mitigate the impact of external risks. Regulatory bodies should continuously optimize regulatory processes, enhance the professionalism of regulators, and ensure the efficient operation of the regulatory system. Furthermore, based on the applicability analysis of this study, the government should strengthen environmental regulation in areas where it is lacking to promote the pace of green innovation and alleviate environmental pressure.

## Supporting information

S1 File (DOCX)
