# Supplementary material for: The role of digital inclusive finance in green innovation
Source: PLoS One. 2024 Dec 23;19(12):e0315598. doi: 10.1371/journal.pone.0315598 (PMC11666025; doi:10.1371/journal.pone.0315598)
Supplement: S1 File — (DOCX) [file pone.0315598.s001.docx]

S1 file

Robustness test

Replacement Strategy for the Dependent Variable

Considering potential delays in the review period from patent application to final authorization and the latent disparities between authorization and application, this study aims to ensure the robustness of our results. We opt to use the number of green patent authorizations processed with the "add one and take the logarithm" method as a new dimension to measure green innovation. As shown in column (1) of S1 Table, the coefficient for DFI reaches 0.0354, indicating a positive relationship at the 1% significance level. This further corroborates that the advancement of digital inclusive finance can notably stimulate firms to pursue green innovation. This is in line with the views of Zhai et al. (2021)[1].

Validation Using Difference GMM Method

To effectively address the endogeneity issue of digital inclusive finance, we employ the Difference GMM method, in accordance with the research approach of Rao et al. (2022)[2]. We use the first and second lag of digital inclusive finance as its instrumental variables, and re-run the regression of equation (1). Regression results from column (2) of S1 Table show that the coefficient for DFI is 0.1810, presenting a positive association at the 1% significance level, maintaining consistency with our previous findings.

Introduction of High-dimensional Fixed Effects for Depth Control

To further control for unobservable factors at the provincial level that might influence the conclusions due to time variations, this study incorporates provincial fixed effects. The regression results revealed in column (3) of S1 Table indicate that the coefficient for DFI is 0.1085, exhibiting a positive effect at the 1% significance level, which aligns with our preceding research outcomes.

S1 Table Robustness test 1

|  | (1) | (2) | (3) |
| --- | --- | --- | --- |
|  | Inno2 | Inno | Inno |
| DIF | 0.0354^***^ | 0.1810^***^ | 0.1085^***^ |
|  | (0.0136) | (0.0341) | (0.0273) |
| Stock | 0.0006^**^ | 0.0025 | -0.0001 |
|  | (0.0003) | (0.0016) | (0.0005) |
| Board | 0.0342 | -0.0871^*^ | 0.0440 |
|  | (0.0226) | (0.0482) | (0.0334) |
| Growth | -0.0019 | 0.0019 | -0.0082^***^ |
|  | (0.0016) | (0.0029) | (0.0024) |
| Size | 0.1387^***^ | 0.0080 | 0.2389^***^ |
|  | (0.0059) | (0.0287) | (0.0086) |
| Age | -0.0188^*^ | -0.7717^***^ | -0.0701^***^ |
|  | (0.0110) | (0.1460) | (0.0163) |
| Wage | 0.0574^***^ | 0.0044 | 0.0977^***^ |
|  | (0.0088) | (0.0288) | (0.0133) |
| State | 0.0016 | -0.0185 | -0.0198 |
|  | (0.0109) | (0.0286) | (0.0161) |
| Power | -0.0630^**^ | 0.2373^**^ | -0.0671 |
|  | (0.0315) | (0.1117) | (0.0468) |
| Roa | -0.0940 | -0.1111 | 0.3136^***^ |
|  | (0.0768) | (0.1321) | (0.1113) |
| LR | -0.0028^**^ | 0.0003 | -0.0101^***^ |
|  | (0.0011) | (0.0025) | (0.0017) |
| Fix | -0.0008^***^ | -0.0006 | -0.0016^***^ |
|  | (0.0003) | (0.0009) | (0.0004) |
| Capi | -0.0094 | 0.0293 | -0.0447^***^ |
|  | (0.0061) | (0.0239) | (0.0092) |
| Con | 0.0002 | 0.0002 | 0.0001 |
|  | (0.0004) | (0.0021) | (0.0005) |
| TBQ | 0.0179^***^ | -0.0177^*^ | 0.0166^***^ |
|  | (0.0044) | (0.0097) | (0.0063) |
| Eco | 0.0007 | -0.0019 | -0.0052^**^ |
|  | (0.0011) | (0.0077) | (0.0026) |
| L.Inno |  | 0.2470^***^ |  |
|  |  | (0.0408) |  |
| _cons | -3.6313^***^ | 1.6213^*^ | -5.4866^***^ |
|  | (0.1585) | (0.8380) | (0.2324) |
| Control | YES | YES | YES |
| Province_FE | NO | NO | YES |
| Industry_FE | YES | YES | YES |
| Year_FE | YES | YES | YES |
| Obs | 11693 | 7662 | 14220 |
| r2_a | 0.1989 |  | 0.2637 |

Analysis of the Core Explanatory Variable and Control Variables Lagged by One Period

Considering that the progress of digital inclusive finance might have a time-lagged impact on firms' green innovation activities, this study re-ran the regression analysis of equation (1) after lagging the core explanatory variable and control variables by one period. As demonstrated in column (1) of S2 Table, the coefficient for DFI is 0.0467. This result is positively significant at the 5% level, confirming that even when accounting for the time-lagged effect of digital inclusive finance, the study's conclusions remain consistent and robust.

S2 Table Robustness test 2

|  | (1) |
| --- | --- |
|  | Inno |
| LDIF | 0.0467^**^ |
|  | (0.0238) |
| LStock | -0.0003 |
|  | (0.0006) |
| LBoard | 0.0643 |
|  | (0.0397) |
| LGrowth | -0.0097^***^ |
|  | (0.0031) |
| LSize | 0.2573^***^ |
|  | (0.0104) |
| LAge | -0.0875^***^ |
|  | (0.0193) |
| LWage | 0.1012^***^ |
|  | (0.0156) |
| LState | -0.0040 |
|  | (0.0190) |
| LPower | -0.0754 |
|  | (0.0559) |
| LRoa | 0.7644^***^ |
|  | (0.1503) |
| LLR | -0.0110^***^ |
|  | (0.0021) |
| LFix | -0.0019^***^ |
|  | (0.0005) |
| LCapi | -0.0594^***^ |
|  | (0.0110) |
| LCon | 0.0003 |
|  | (0.0006) |
| LTBQ | 0.0192^**^ |
|  | (0.0081) |
| LEco | -0.0029 |
|  | (0.0020) |
| _cons | -5.7647^***^ |
|  | (0.2780) |
| Control | YES |
| Industry_FE | YES |
| Year_FE | YES |
| Obs | 10644 |
| r2_a | 0.2631 |

Exploration of the Instrumental Variable Regression Method

Qian et al. (2020)[3] noted that the evolution of digital finance inherits certain traits from traditional finance and has a complex bidirectional causal chain with economic growth. The myriad factors influencing economic growth also suggest that the control variables this study relies upon might not fully eliminate the emergence of omitted variables. To alleviate potential endogeneity issues, we further employ the instrumental variable method.

Huang and her team (2019)[4] inferred from the development history of internet technology in China that its rapid growth began during the proliferation phase of landline telephones. Before the widespread use of landlines, people primarily relied on the postal system for communication. Hence, the advancement of the postal system likely presaged a region's promising future in digital inclusive finance development. The development of the post office can thus be regarded as an influential factor in digital inclusive finance growth. Moreover, historical data indicates that past per capita postal and telecommunication business volumes have minimal impact on current macroeconomic growth (Qian Haizhang et al., 2020). Thus, we chose the historical per capita postal and telecommunication business volume (IV) as an instrumental variable, meeting the requirements of relevance and exclusivity. Following this line of reasoning, we utilize the 1984 per capita postal and telecommunication business volume for various Chinese cities (IV) as an instrumental variable for measuring the development of city digital inclusive finance.

According to the 2SLS regression results, S3 Table displays regression outcomes from two stages. Column (1) provides the results from the first-stage regression analysis, while column (2) presents the second-stage data. From column (1), the coefficient for IV is 0.0164, which is positive and significant at the 1% level, indicating the absence of a weak instrument problem. This confirms that the 1984 per capita postal and telecommunication business volume indeed had an impact on current digital inclusive finance development. In column (2), the coefficient for DFI is 0.1627, reflecting a positive relationship significant at the 10% level, suggesting that the advancement of digital inclusive finance stimulates green innovative behavior. After further endogeneity testing, the study's conclusions remain validated.

S3 Table Robustness test 3

|  | (1) | (2) |
| --- | --- | --- |
|  | DIF | Inno |
| IV | 0.0164^***^ |  |
|  | (0.0006) |  |
| DIF |  | 0.1627^*^ |
|  |  | (0.0932) |
| Stock | 0.0007^***^ | 0.0001 |
|  | (0.0002) | (0.0005) |
| Board | -0.0320^**^ | 0.0602^*^ |
|  | (0.0139) | (0.0348) |
| Growth | -0.0014 | -0.0089^***^ |
|  | (0.0010) | (0.0025) |
| Size | -0.0048 | 0.2474^***^ |
|  | (0.0035) | (0.0088) |
| Age | -0.0448^***^ | -0.0731^***^ |
|  | (0.0067) | (0.0174) |
| Wage | 0.0632^***^ | 0.0917^***^ |
|  | (0.0055) | (0.0150) |
| State | 0.0121^*^ | -0.0241 |
|  | (0.0067) | (0.0168) |
| Power | 0.0175 | -0.0948^*^ |
|  | (0.0196) | (0.0491) |
| Roa | 0.0495 | 0.3770^***^ |
|  | (0.0462) | (0.1151) |
| LR | -0.0018^**^ | -0.0113^***^ |
|  | (0.0007) | (0.0018) |
| Fix | 0.0007^***^ | -0.0018^***^ |
|  | (0.0002) | (0.0004) |
| Capi | 0.0157^***^ | -0.0527^***^ |
|  | (0.0038) | (0.0095) |
| Con | 0.0009^***^ | -0.0003 |
|  | (0.0002) | (0.0006) |
| TBQ | 0.0027 | 0.0153^**^ |
|  | (0.0026) | (0.0065) |
| Eco | 0.0335^***^ | -0.0086^**^ |
|  | (0.0007) | (0.0044) |
| Control | YES | YES |
| Industry_FE | YES | YES |
| Year_FE | YES | YES |
| Obs | 13499 | 13499 |
| r2_a |  | 0.1085 |

References

1. Zhai HY, Lewis. Research on the relationship between the development of digital finance, financing constraints, and corporate green innovation. Sci Technol Prog Countermeas. 2021;(17):116-124.
2. Rao P, Wu Q. The impact of digital inclusive finance on enterprise total factor productivity. Stat Decis. 2022;(16):142-146. doi:10.13546/j.cnki.tjyjc.2022.16.028.
3. Qian HZ, Tao YQ, Cao SW, Cao YY. The theory and empirical evidence of China's digital finance development and economic growth. Quant Econ Tech Econ Res. 2020;(06):26-46. doi:10.13653/j.cnki.jqte.2020.06.002.
4. Huang QH, Yu YZ, Zhang SL. Internet development and manufacturing productivity improvement: Internal mechanism and China's experience. China Ind Econ. 2019;(08):5-23. doi:10.19581/j.cnki.ciejournal.2019.08.001.
